# Davis’ Medical History, Chapter II

**Published:** 1850-09

**Authors:** N. S. Davis

**Affiliations:** Prof. of Physiology and Pathology in Rush Medical College


THE NORTH-WESTERN
MEDICAL AND SURGICAL JOURNAL.

VOL. III.] SEPTEMBER, 1850. [No. 3.

### Part 1.—Original Communications.

### CHAPTER II.—CONTINUED.

*History of the Medical Profession from the first settlement of the British Colonies in America, to the year* 1850.—By N. S. DAVIS, M. D., Prof. of Physiology and Pathology in Rush Medical College.

[Copy Right Secured.]

The period of study was extended to four years, with a deduction of one year in favor of those who had graduated at some literary College. Each candidate was also required to furnish the examining officers with a certificate of the time he had studied, verified by the oath of his preceptor; and his license was to be filed in the Clerk's Office of the County where he commenced practice.

Those who had received the Degree of Bachelor or Doctor of Medicine from a Medical College, were permitted to practice on filing a copy of their Diploma in the County Clerk's Office, without any further examination. The great defect in this law, consisted in the fact, that the certificate, setting forth the time of study &c., and verified by the oath of the preceptor, became the only test of qualifications for practice; it being only necessary to exhibit this to the proper officer, and file it in the office of the County Clerk. It, however, remained with but slight amendment until 1806, and its provisions embraced the whole State.

The Medical Society which had existed in New Jersey since 1766, was regularly incorporated by the Legislature of that State, in 1790, under the name of the "Medical Society of the State of New Jersey." The act of incorporation conferred the power to appoint Censors for the purpose of examing and licensing candidates for permission to practice in that State; also to establish district or county societies, whose delegates were to constitute the Parent or State Society. The term of study required, and all the regulations adopted, were very similar to those prescribed by the law of 1797, in New-York. The Medical Society of South Carolina was incorporated in 1794, but no provision for examining and licensing candidates for admission into the profession, was made until 1817.

In 1799, the "Medical and Chirurgical Faculty of the State of Maryland" was incorporated, with power to elect "by ballot, twelve persons of the greatest medical and chirurgical abilities in the State, who shall be styled the Medical Board of Examiners for the State of Maryland." It was the duty of this Board, "to grant licenses to such medical and chirurgical gentlemen, as they, either upon a full examination, or upon the production of Diplomas from some respectable college, may judge adequate to commence the practice of the medical and chirurgical arts."

Under a supplementary act passed in 1801, the board of examiners required all graduates of medical colleges, as well as others, to apply for and obtain a license before being authorized to practice. The penalty for practicing in violation of the foregoing provisions, was fifty dollars for each offence, to be recovered in the County Court where the offender may reside; and the Judges of those courts were directed to give the several acts relating to medicine and surgery, annually, in charge to their respective Grand Juries.* Every person licensed by the examining board, was, by virtue of such license, constituted a member of the State Society. It is thus

*See Act of Incorporation, Supplementary Acts, &c., 18 mo. Balt, 1822.

seen that Maryland was, not only, among the earliest to enact laws to protect her citizens against the inroads of ignorance and empiricism, but also that her laws relating to this subject, were at once simple and effectual. By the foregoing, it will be seen that six of the original thirteen States had recognized their right and duty to legislate on the subject of medical education and practice during the first twenty years after the Revolutionary war.

Concerning the general condition of the profession at this time, we have the testimony of some of its most distinguished members, as well as other authority of a reliable character. In the peamble to the law passed by the Legislature of New York, in 1792, it is stated that "many ignorant and unskillful persons presume to practice physic and surgery within the city and county of New York, to the great detriment and hazard of the lives and limbs of the citizens thereof," &c. And the venerable Dr. John Stearns, in speaking of this period, says: "those who witnessed the original and progressive settlement of the northern and western sections of this (New York) State, since the year 1791, will recognize the mania that infatuated the emigrants from the east, and the ambitious projects formed by those who assumed the title of Doctor. Many who had never read a volume in medicine, were suddenly introduced to an extensive practice, and to a reputation of such imposing authority as to control the opinions of their superiors in science, and to prescribe rules of practice for their government. Consultations were generally distinguished for gross controversies at the *bed*-side of the patient, whose health and life were often immolated to the ignorance, prejudices, or discordant theories of the contending physicians. Their skill was generally graduated by their ability to magnify the cures they had made."* Again the same writer, in an Anniversary Address read to the Medical Society of the State of New-York, in 1818, says: "Science was almost exclusively limited to our popu-

*See United States Medical and Surgical Journal, No. 16, page 122.

lous cities. With a few honorable exceptions in each county, the practitioners were ignorant, degraded, and contemptible. Without any system of principles, their practice was desultory and empirical." Although the foregoing extracts were written solely in reference to the profession in the State of New York, yet they will apply with sufficient correctness to all parts of the Union.* As we have seen, only three or four States had attempted to place any positive restrictions on irregular or empirical practice; and not more than 300 students were annually found in attendance on the three or four Medical Colleges that were in active operation during this period. And the whole number of graduates did not average 15 annually. Still this was an important era in the history of American Medicine. It was the period of emanicipation, if we may so speak, from obedience to European theories and dependence on European institutions. It was the forming period of a most difficult profession, in a new and widely extended country, and while the great mass were neither learned in science nor skilled in practice, there was to be found in every State a few who had attained prominence in both.

The names of John Warren, Benj. Waterhouse, Richard Bayley, Samuel Bard, Edward Miller, Samuel L. Mitchell, Wright Post, Nicholas Romayne, Peter Middleton, John Jones, Benj. Rush, Benj. S. Barton, William Shippen, W. P Dewees, John Mitchell, Lionel Chalmers, J. Moultrie, and several others, will ever constitute a bright galaxy on the pages of American medical history. Many of these were not only learned, but they were characterized by a bold free spirit of inquiry, which soon broke through the theoretical dogmas of that day, snd did more by their writings to overthrow the

*The following preamble to a law passed in 1736, will show something in regard to the state of medicine in Virginia.

"Whereas, the practice of physic in this colony, is most commonly taken up and followed by surgeons, apothecaries, or such as have only served apprenticeships to those trades, who often prove very unskilful in the art of a physician," &c.—[See Herring's Statutes of Virginia, Vol. 4th.

absurdities of the Bœrhaavian school, than any equal number of their contemporaries on the other side of the Atlantic. In regard to the medical theories or opinions that prevailed at the period which closes this chapter, we are told by Dr. Rush that "the system of Bœrhaave long ago ceased to regulate the practice of Physic. It was succeeded by the system of Cullen. In the year 1790, Dr. Brown's system of medicine was introduced and taught by Dr. Gibbon. It captivated a few young men for a while, but it soon fell into disrepute." In the year 1790, Dr. Rush promulgated some principles which he regarded as the foundation of a new system in medicine, of which the following is a summary in his own language. "This system rejects the nosological arrangement of diseases, and admits only of a single disease, consisting in different forms of morbid excitement, induced by irritants acting upon previous debility. It rejects, further, an undue reliance upon the powers of nature, and teaches, instantly to wrest the cure of all violent and feeble diseases out of her hands; and lastly it rejects prescriptions for the names of diseases; and by directing their application wholly to their forming and fluctuating states, derives from a few active medicines all the advantages which have been in vain expected from the numerous articles which compose European treatises upon the materia medica." But notwithstanding the plausability of the doctrines promulgated by Dr. Rush, the system of Cullen still held sway in the minds of the majority of practitioners throughout the country. He, however, struck an effectual blow against those cumbersome and arbitrary nosological arrangements which had so long served to mislead and obscure rather than enlighten the professional mind. These doctrines were also chiefly instrumental in causing the general adoption, in this country, of a much more active and depletory, or antiphlogistic, system of treating diseases than had previously prevailed—a system of treatment, indeed, which still retains the confidence of a large portion of the profession. It is true that his opinions and recommendations in regard to blood-letting, were sometimes extravagant, and on that account, have more recently met with severe censure from the most eminent members of the profession.* But before we indulge in a too sweeping condemnation, and attribute to Dr. Rush the origin of all the evils which have resulted from a too prodigal indulgence in the use of the lancet, we should first inquire into the particular character of the diseases prevalent in the community during the period of his own observations; second, whether that character has since undergone any change; and third, whether the evils alluded to were owing to his erroneous doctrines, or to the application of them, unchanged, by his successors, to diseases essentially modified in their character. If we call to mind the habits of the people of Philadelphia just previous to the Revolutionary war, as already briefly detailed in the preceding chapter, and remember that upon such habits supervened the strong and universal political and warlike excitement of the Revolution itself, accompanied by far greater abstemiousness and physical hardihood, we shall not fail to see a combination of causes well calculated to give a highly stenic or inflammatory character to all such diseases as should prevail at that period. Neither did the influence of these causes cease for several years after the close of the war. But we are not left simply to inferences in regard to the nature of the diseases of that period. Dr. Rush says, in speaking of the diseases of Philadelphia: "Fevers have assumed several new forms since the year 1766. The mild Billious Fever has gradually extended itself over the whole city.— * * * In the years 1791 and 1792, it assumed an inflammatory appearance, and was accompanied, in many cases, with hepatic affections. It appeared in 1793 as an epidemic, in the form of what is called Yellow Fever, in which form it has appeared, in sporadic cases, or as an epidemic,

*See Beck on Bloodletting, in the Young Subject.

nearly every year since. During the reign pf this high grade of Billious Fever, mild Intermittents and Remittents, and the chronic or nervous forms of the summer and autumnel Fever, have nearly disappeared. Inflammations and obstructions of the liver have been more frequent than in former years, and even the Pneumonias, Catarrhs, Intercurrent, and other Fevers of the Winter and Spring months, have all partaken more or less of the inflammatory and malignant nature of the Yellow Fever." Whoever will compare the most accurate accounts which are left on record, of the diseases that prevailed between 1780 and 1806, with the phenomena of diseases prevalent in the same cities at the present time, will certainly find little difficulty in recognizing a marked difference in their character. How much similarity is there between the slow nervous or typhoid Fevers which occur in all our large cities, or the more fully formed Typhus of the emigrants and poorer classes, and the Congestive, Inflammatory, and rapidly progressing Fevers of the period embraced in this chapter of our history? And what right have we to judge of the propriety of active depletion in the latter, by our knowledge of its effects in the former? No more, certainly, to use an apt comparison of Rush, than we have to consult the almanacks of 1803 for the monthly phases of the moon of the present year. The character of diseases, however, not only varies at different periods of time, but in different localities, also.

Thus, I have been intimately acquainted with the practice of a physician, who, during a ten year's residence and extensive practice in a rugged agricultural district, was uniformly in the habit of using the lancet freely in the first stage of all febrile and inflammatory attacks occurring in his district, where the patient had been previously in good health, and with the most decided and uniform success. But does any one imagine that the same results would follow the application of this practice among the delicate and fashionable classes on the one hand, or the ill-fated and uncared for poor on the other, which together make up so large a share of the population of all our large cities? The truth is that professional men are too apt, though perhaps unconsciously, to think and speak of disease as a positive *entity*—as a something with well defined and uniform characteristics. And hence they are continually prone to condemn their predecessors for using in a given disease whatever their own experience shows to be inapplicable to the disease, called by the same name, as it occurs in their day. A full recognition of the fact that disease is only a morbid condition of previously healthy functions or structures, would show every thinking mind, that the special tone or character of any disease must depend entirely on the tone or character of the previous standard of health and the quality of the exciting causes. Every intelligent physician fully recognizes this principle in its application to different cases of the same disease occurring in his own practice, but often forgets it when comparing his practice with that of his predecessors, or even with that of his cotemporaries of a distant, and, perhaps, essentially different locality. Hence he is apt to condemn others for what is really his own fault, viz: an attempt to apply the practice of another to a disease, merely because it is called by the same name, without reference to the question whether it prevails in a similar locality, among people of the same habits, and arising from causes of the same quality and grade of intensity. If we feel the force of these thoughts, while we remember that Dr. Rush came upon the stage of action at a period when the professional mind had scarcely yet divested itself of the humoral and expectant notions of treatment inculcated by the Bœrhaavian school, and that he lived in the midst of opposition, we shall feel much more disposed to excuse than censure his seeming extravagancies, and to inquire seriously whether we have as rigidly examined the diseases of our day as he did those of his.

The views of Dr. Rush, in reference to bleeding, were warmly seconded by Drs. Griffitts, Dewees, Physick, &c., while they were strenuously opposed by many others. In 1806, Dr. W. P. Dewees published his elaborate and interesting Inaugural Thesis, "on the means of moderating or relieving pain during the process of Parturition." In this paper, he maintained the doctrine that "*pain* was an accidental or morbid sympton of labor—the result of artificial modes of living and treatment." The remedy which he proposed and successfully practiced through a long series of years, was copious blood-letting. The same doctrines and practices had been ably defended by Dr. Peter Miller, in an inaugural thesis, published in Philadelphia, in 1804; but Miller had himself attended the private lectures of Dr. Dewees, and doubtless derived from him the views set forth in his essay. The views of Dr. Dewees on this subject met the cordial approbation of both Rush and Shippen; the latter of whom is said to have declared that the Thesis of 1806 "marked an *era* in the history of medicine." It is true, that bleeding during parturition had long been practiced in different countries of Europe, and even by the midwives of Genoa; but its advocacy for the *purposes* mentioned in the paper of Dewees, and to the copious extent practiced by him and his followers, was new, and therefore, strictly American in its origin. The reader will not fail to notice, that the doctrine here advocated in reference to the nature of the *pains* of labor, is directly the opposite of that inculcated by a distinguished professor and author in the same city, now occupying the front rank among the obstetricians of the present age.—Neither will he fail to notice that Dewees practiced copious bleeding for the same purpose, and, in many instances, enforced its necessity with the same arguments that are now used by Simpson and others in favor of anesthetic agents.

The following paragraph from Rush's celebrated defence of blood-leting is worthy of notice as showing that the very idea, the practical accomplishment of which constitutes one of the prominent glories of medicine in our day, was fully present in the minds of such men as Rush and Dewees.

"I have expressed a hope in another place,"* says Dr. Rush, "that a medicine would be discovered that should suspend sensibility altogether, and leave irritability or the powers of motion, unimpaired, and thereby destroy labor pains altogether. I was encouraged to cherish this hope, by having known delivery to take place, in one instance, during a paroxysm of Epilepsy, and having heard of another, during a fit of drunkenness, in a woman attended by Dr. Church, in both of which there was neither consciousness, nor recollection of pain." Here the drunken woman, doubtless, presented as fair a case of anæsthesia as though she had inhaled ether or chloroform. Dr. Rush even goes further than Dewees, and represents the uterus as in a diseased state during the period of pregnancy and parturition. He says: "In *pregnancy*, the uterus is always affected with that grade of of morbid action which I formerly *called inflammation.* This is evident from its exhibiting all its usual phenomena in other parts of the body." Again, he says:—

"*Parturition* is a higher grade of disease than that which takes place in pregnancy. It consists of convulsive or chronic spasms in the uterus, supervening its inflammation, and is accompanied with chills, heat, a quick, full, tense, or a frequent and depressed pulse, and great pain." And after alluding to one of the most important points discussed in the well known correspondence between Professors Meigs and Simpson, relative to the propriety of obviating the pains of labor by the use of anæsthetic agents, he adds: "I was induced to believe that pain does not accompany child-bearing by an immutable decree of Heaven." Hence, he fully accords with Dr. Dewees in recommending free and even copious blood-letting to relieve the pains of pregnancy and parturition unless it should be contra-indicated by previous inanition or other causes of languor and enfeebled circulation.

*See Medical Repository, Vol. VI.

Perhaps no one thing has served to render the period now under consideration more conspicuous, than the warm and interesting controversy which was maintained in reference to *contagion*, especially as applied to the origin and spread of Yellow Fever. The repeated visitations of this disease even as far north as New York and Philadelphia, during the last part of the eighteenth and the first few years of the nineteeth centu y, caused it to be a subject of absorbing interest. And as it was always confined to commercial towns and cities located near the sea, or the islands in the Indies, it was very natural that the earliest observers should attribute it to importation, and regard it, therefore, as a highly contagious disease. The correctness of this view was soon called in question, however; and though supported by such men as Lining, of South-Carolina, Mitchell, of Virginia, and Hosack, of New York, yet it rapidly lost its hold on the professional mind of this country, under the most searching and able opposition of Dr. Rush and a few others. And few questions are considered better settled at the present day than that Yellow Fever is not contagious. Those who desire to examine this interesting subject with care, will do well to consult the early volumes of the New York Medical Repository, the two medical journals then published in Philadelphia, and also the Medical Inquiries and and observations of Dr. Rush. Before closing this chapter, we must allude to two important historical mistakes made by eminent medical men abroad. The first is by Dr. John Armstrong, who attributes to Dr. Robert Hamilton the merit of first introducing the use of Mercury as a remedy in inflammatory diseases. The latter gentleman, in his own account of the remedy, says his attention was first called to it by a medical officer of the army, in 1764.—Whereas, we have seen in the preceding chapter, what Dr. J. B. Beck has so fully presented in his little volume on Infant Therapeutics, that Mercury in the form of Calomel was used in a malignant epidemic sore throat by Dr. Douglass, of

Boston, as early as 1736. It was still more extensively used and recommended by Dr. James Ogden, of Long Island, in 1749 ; and it was very successfully introduced into the practice of the profession in Philadelphia about the same time by Dr. Thomas Bond. And Dr. Rush says, in speaking of the period which intervened between 1760–66, "Mercury was in *general* use in the years that have been mentioned," We thus see that the practice of which Dr. Armstrong speaks, was already in general use in the American colonies even prior to the time it was first suggested to Dr. Hamilton. The second mistake to which we allude is that of Dr. Stokes, in his valuable treatise on diseases of the chest, where he gives Dr. Cheyne the credit of having first introduced the practice of giving Tartar-Emetic in the Cynanche Trachialis or croup. The first publication on the subject, by Dr. Cheyne, was in 1801. But Dr. Richard Bayley, of New York, had accurately pointed out the great value of this remedy in the same disease, in a letter to Dr. Wm. Hunter, published in New-York city in 1781—just twenty years previous to the publication of Dr. Cheyne. Dr. Bayly is, not only, fully entitled to the credit given by Dr. Stokes to Dr. Cheyne, but it is to him that the profession are also indebted for having first pointed out the true inflammatory nature of the disease here referrred to.* It was also during the last part of the eighteenth century that Dr. Thomas Cadwallader, of Phila., first introduced the practice of treating the "dry gripes," or Colica Pictonum and Bilious Colic with *anodynes* until the pains and spasms were allayed, and then moving the bowels only by gentle laxatives. The almost universal custom had been to give the most active and drastic purgatives, which often served only to increase the muscular contraction of the intestines instead of relieving the patient. The treatment practiced by Dr. Cadwallader, was afterwards adopted and highly recommended by Dr. Warren, of London.

The medical writings of this period seem to have been confined almost wholly to the pages of the three medical journals which had been established in New York and Philadelphia, and to here and there a pamphlet or a paper read before some organized society. Indeed, I have not been able to find a single volume on any branch of medical science or practice, published by an American physician during the first twenty years after the close of the Revolutionary war.

And yet, as we have already seen, it was a period in our history, which was graced with some of the noblest and most active minds ever devoted to the cultivation of medical science. But the art of *mere book-making*, which has been brought to such perfection in this our day, was little known to our professional ancestors.

### CHAPTER III.

### HISTORY OF THE PROGRESS OF MEDICAL EDUCATION FROM 1806 TO 1850.

To those who limit the means of education to the preceptor's office and the college halls, and the period of its acquirement to the season of pupillage (and there are unfortunately many such), much of the matter contained in this work may appear irrelevant or superfluous. But it is unnecessary for us to state that we attach to the word a far more liberal and comprehensive meaning. Whatever increases the enterprize, stimulates the spirit of philosophical investigation, or adds an item to the stock of knowledge possessed by the profession, or whatever elevates it in the great scale of social being, is as truly a part of its education as is the study of its text-books, and the frequenting of its schools. The latter may indeed constitute the foundation, but many other things are required to complete the superstructure of a fair medical education. And among those other things, none are of greater importance than well organized Associations, admitting of frequent communications and free interchange of thought among their members. Such Associations not only elicit observations, stimulate investigations, and save from oblivion numberless facts, but they counteract the selfish feelings of iudividuality—they diffuse knowledge—they elevate thc social feelings—and they embody and generalize facts that otherwise would remain isolated and useless. In this respect, the commencement of the period embraced in this chapter, forms an important era in the history of the medical professton in this country. For though several medical societies had been duly incorporated, and some of them continued in active operation for a quarter of a century, yet they were almost wholly confined in their influence to a few of the larger cities. Hence, as intimated near the close of the last chapter, the great mass of the profession were alike unsocial and ungoverned by ethical laws, and consequently without harmony of action or true dignity of professional character. This condition of things was fully appreciated by a few enlightened members of the profession in Saratoga County, New York, so early as 1796, during which year several articles on the subject of a local medical Association appeared in the newspapers of that county. These articles we believe were from the pen of Dr. John Stearns, then residing in that section of the State. So much attention was awakened that a County Society was soon after formed, containing twenty-one members; but so discordant were their feelings and modes of thought, that the same year saw both its organization and dissolution. This failure, however, by no means deterred the enlightened advocates of medical organization from further efforts. In Nov., 1805, another meeting of the physicians of Saratoga county was held, and a resolution adopted, inviting their medical brethren in Washington and Montgomery counties to join them in the formation of a Society to be incorated by the State Legislature. The committee appointed to carry the resolution into effect, consisted of Drs John Stearns, Wm. Patrick, and Grant Powell. The committee issued a circular to the physicians of Washington and Montgomery, in response to which they sent delegates to the adjourned meeting at Ballston, on the 16th of January, 1806. A memorial to the Legislature, asking for an act of incorporation, was reported at this meeting, adopted, and a committee appointed to carry it into effect. This committee consisted of Dr. John Stearns, of Saratoga, Dr. Alex. Sheldon, of Montgomery, and Dr. Asa Fitch, of Washington. Fortunately the two first members of the committee had been also elected members of the popular branch of the Legislature, and one of them, Dr. Shelden was elected Speaker of the House.

It will be observed that the memorial contemplated nothing more than the passage of a law in reference to the three counties named, but the enlightened committee to whom it had been committed, in presenting it to the Legislature, took a wider survey of the wants of the profession, and asked for for a law applicable to every county in the State. The memorial to the House of Assembly was referred to a committee of five, a majority of whom were medical men; and they consequently soon matured and reported a general act of incorporation, not only applicable to each county in the State, but also providing for a State Society composed of delegates from the several county Associations. But politicians so magnified the dangers to which the State would be exposed from the incorporation of more than forty Associations of physicians within its limits, that despite of the most earnest support of both committees, the rejection of the proposed law by a large majority was considered almost certain. Owing, however, to the very able and eloquent advocacy of the bill at this critical period, by the Hon. Wm. W. Van Ness, it finally received the sanction of the Legislature on the 4th of April, 1806.

This law authorized the legally qualified physicians and surgeons of each county to form themselves into a Society, named after the county in which it was formed, with power to choose officers, make all needful rules for the government of its members, and appoint a Board of Censors to examine and license all the applicants for admission into the profession in their respective counties. But no one could be admitted to an examination until he had given evidence of having studied three years with some practitioner, and had arrived to the age of twenty-one years. A State Medical Society was also provided for, to be composed of one delegate from from each County Society, and such permanent members as the Society should from time to time elect, not exceeding two in any one year. It was required to meet annually at the capitol in the city of Albany to elect officers and transact such other business as the interests of the profession should require. It was also required to divide the State into four medical districts and appoint a Board of Censors for each, whose duty it was to examine all candidates for license to practise medicine and surgery who should present themselves after having studied the required length of time. The law also forbid any to enter the profession and collect pay for their services without first procuring either a license from a County or State Society, or a diploma from some regularly organized medical college. Candidates who might be rejected by the County Boards had the right to appeal to the Censors of the State Society for another examination ; but not *vice versa*.

Within two years after the passage of this law nearly every county in the State had its regularly organized medical Society, with its Board of Censors and library.

The first meeting of the State Society was held at the capital in Feb., 1807, and completed its organization according to the provisions of the law. Thus two great and all important objects were accomplished viz : a thorough organization of the profession in a manner most favorable to its advancement and elevation, and the provision for having all candidates exam-

